# Melatonin and Exercise Counteract Sarcopenic Obesity through Preservation of Satellite Cell Function

**DOI:** 10.3390/ijms24076097

**Published:** 2023-03-23

**Authors:** Sakulrat Mankhong, Sujin Kim, Sohee Moon, Jae-Seon Lee, Eun-Jeong Cho, Hyo-Bum Kwak, Dong-Ho Park, Ji-Kan Ryu, Ju-Hee Kang

**Affiliations:** 1 Department of Pharmacology, College of Medicine, Inha University, Incheon 22212, Republic of Korea; 2 Research Center for Controlling Intercellular Communication, College of Medicine, Inha University, Incheon 22212, Republic of Korea; 3 Program in Biomedical Science and Engineering, Inha University, Incheon 22212, Republic of Korea; 4 Department of Molecular Medicine, Inha University, Incheon 22212, Republic of Korea; 5 Department of Kinesiology, Inha University, Incheon 22212, Republic of Korea; 6 Department of Urology, College of Medicine, Inha University, Incheon 22212, Republic of Korea

**Keywords:** melatonin, exercise, sarcopenia, skeletal muscle, aging, cellular senescence

## Abstract

Sarcopenic obesity (SO) is characterized by atrophic skeletal muscle impairment (sarcopenia) and obesity, which is associated with adverse outcomes of morbidity and mortality in elderly people. We investigated the effects of melatonin and exercise training on SO in 32-week-old senescence-accelerated mouse-prone-8 (SAMP8) mice fed a normal diet or a high-fat diet for 16 weeks. Melatonin, exercise, or melatonin and exercise for 8 weeks displayed reductions in the SO-induced impairment of skeletal muscle function and atrophy. Specifically, a decrease in mitochondrial calcium retention capacity in skeletal muscles observed in the HFD-con group was attenuated in melatonin and/or exercise intervention groups. More importantly, HFD-con mice displayed a lower number of Pax7+ satellite cells (SCs) and higher expression of p16^ink^ than P8ND mice, which were attenuated by melatonin and/or exercise interventions. The cellular senescence in SC-derived primary myoblasts from HFD-con mice was significantly attenuated in myoblasts from the melatonin and/or exercise groups, which was reproduced in a senescence model of H_2_O_2_-treated C2C12 myoblasts. Our results suggest that melatonin and exercise training attenuate SO-induced skeletal muscle dysfunction, at least in part, through preserving the SC pool by inhibiting cellular senescence and attenuating mitochondrial dysfunction.

## 1. Introduction

Aging is a well-known risk factor that is associated with the progressive decline of skeletal muscle mass and function, commonly referred to as sarcopenia. Elderly people who are simultaneously sarcopenic and obese are particularly susceptible to comorbidities, which can lead to long-term disability [1]. Although several molecular mechanisms—such as proteostasis imbalance, cellular senescence, inflammation, oxidative stress, and mitochondrial dysfunctions—have been implicated in sarcopenic obesity (SO) [2], the precise underlying mechanisms remain unclear. During aging, the number of satellite cells (SCs) decreases, and they become dysfunctional and unable to regenerate effectively [3,4]. The occurrence of SC senescence in aged skeletal muscle contributes to the depletion and dysfunction of the SC pool [4,5]. In addition, skeletal muscle SCs can be impacted negatively by obesity [6]. Furthermore, an age-related loss of skeletal muscle mass and function may partially result from cumulative episodes of incomplete muscle repair. Therefore, it is assumed that inhibition of pre-senescent factors can improve SCs’ function in aged muscle, although few anti-sarcopenic drugs have been developed [7]. 

Melatonin has many biological actions, including antioxidant, anti-inflammatory, and antitumor effects [8], and pineal production and plasma levels of melatonin reduce progressively with aging [9], suggesting a crucial role in the aging process and age-related diseases [10]. Interestingly, urinary levels of melatonin are inversely associated with the prevalence of sarcopenia in postmenopausal women [11], as well as being correlated with muscle strength in the general elderly population [12]. Considerable evidence supports the beneficial effects of melatonin on skeletal muscle function in acute traumatic injury or metabolic disease, where melatonin restores muscle regeneration [13] or prevents mitochondrial dysfunction [14]. It is established that exercise training can reduce the body’s fat mass and improve skeletal muscle mass and function [15,16]. Therefore, investigating the possible effectiveness and underlying mechanisms of combining exercise and melatonin supplements in combating SO in the elderly population is of interest to assess the possible additional role of melatonin in counteracting SO [17].

In this study, we tested the hypothesis that melatonin and exercise training would mitigate the loss of skeletal muscle mass and function in an in vivo model of SO. We used an aged, senescence-accelerated mouse-prone-8 (SAMP8) strain fed a high-fat diet (HFD) and in vitro myoblast cultures to investigate the potential preventive roles of melatonin administration and/or exercise training against SO, and we explored the underlying mechanisms of the observed benefits.

## 2. Results

### 2.1. Melatonin and Exercise Counteract SO in HFD-Fed SAMP8

**Figure 1:**
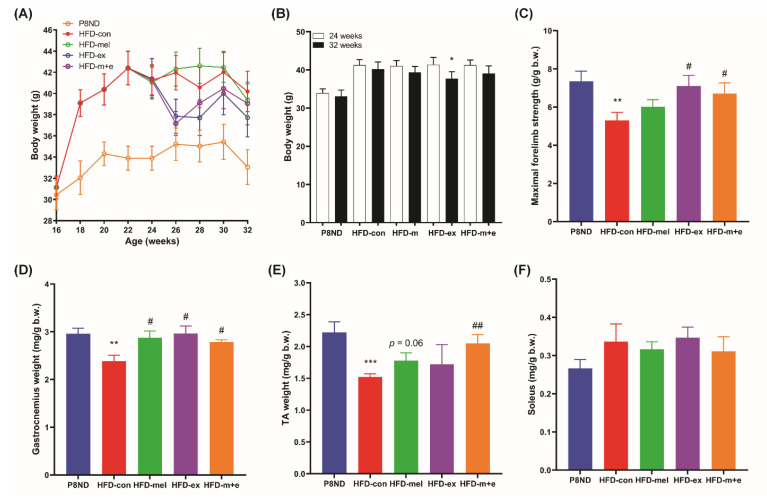
Melatonin and exercise training protected against the reduction in muscle weight and muscle strength in high-fat diet (HFD)-fed SAMP8 mice. Changes in body weight (**A**) during the experiment (16–32 weeks of age) and (**B**) after the experiment (n = 9–10); * *p* < 0.05 vs. 24 weeks by paired *t*-test. (**C**) Maximal forelimb strength and (**D**–**F**) relative muscle weight adjusted by body weight; ** *p* < 0.01, *** *p* < 0.001 vs. P8ND, ^##^ *p* < 0.01, ^#^ *p* < 0.05 vs. HFD-con, by unpaired *t*-test. Data are presented as the mean ± SD.

Mice fed an HFD (HFD-con) displayed a significantly higher body weight than mice in the normal diet (P8ND) group. A comparison of body weight at the age of 24 weeks revealed that exercise training for 8 weeks (HFD-ex group)—but not melatonin (HFD-mel group) or combined intervention (HFD-m+e group)—significantly reduced body weight (Figure 1A,B). At the end of the experiments, we could not detect any noticeable adverse events or signs of abnormal gross morphology in the vital organs in any of the groups. As SO encompasses age- and obesity-related loss of muscle mass and strength, we examined the impacts of melatonin and exercise training on the SO-associated decline in muscle mass and strength. At the end of the experiment, the maximal forelimb grip strength in the P8ND and HFD-con groups was significantly lower than before the intervention (and HFD–con < P8ND), while grip strength in the HFD-ex and HFD-m+e groups was comparable (Figure S1). However, the HFD-mel group displayed a trend of attenuating the decline in strength produced by HFD (+13.38% vs. HFD-con), and the HFD-ex (+33.95% vs. HFD-con) and HFD-m+e groups (+26.48% vs. HFD-con) showed significant improvements in grip strength (Figure 1C). The relative weights (adjusted by body weight) of the white gastrocnemius (WG) and tibialis anterior (TA) muscles in the HFD-con group were significantly lower than those in the P8ND group. The relative weights of the WG muscle in all intervention groups and the TA muscle in the combined intervention group were significantly higher than those in the HFD-con group, but the TA muscle weights in the HFD-mel and HFD-m+e groups were not significantly altered (Figure 1D,E). However, there were no significant differences in soleus muscle weights between the different groups (Figure 1F). A significant elevation in fasting blood glucose levels was observed in HFD-fed mice, which was significantly attenuated by the melatonin and/or exercise interventions. The HFD-con mice also displayed significantly higher plasma levels of leptin and resistin, along with significantly lower levels of adiponectin, compared with those in the P8ND group, but these were not restored by melatonin and/or exercise training (Supplementary Table S2).

### 2.2. Melatonin and Exercise Training Affect the Morphology of the Soleus Muscle

**Figure 2:**
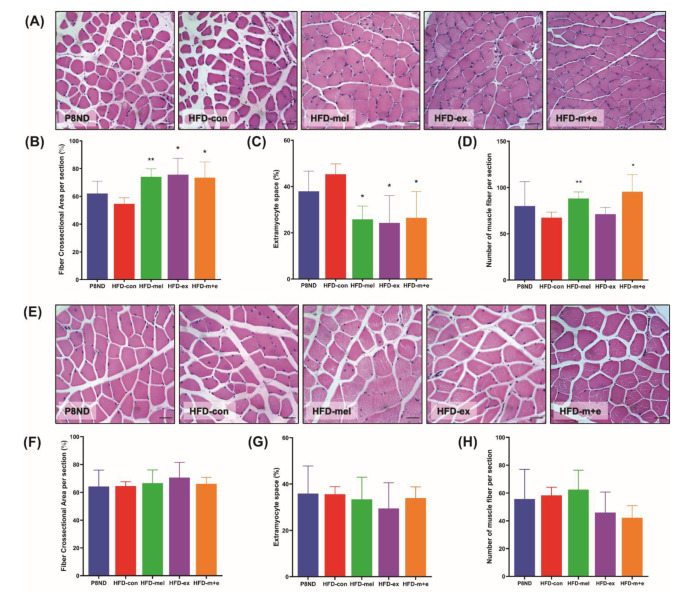
Effects of melatonin and exercise training on skeletal muscle morphological changes: Representative hematoxylin and eosin staining illustrating the effects of melatonin and/or exercise training on myofiber morphology in the (**A**) soleus and (**E**) white gastrocnemius muscles. Bar graphs illustrate (**B**,**F**) the percentage of muscle fiber cross-sectional area (CSA), (**C**,**G**) the percentage of extramyocyte space, and (**D**,**H**) the number of muscle fibers per section in the soleus and white gastrocnemius muscles, respectively. Data are the mean ± SD (n = 4–5 mice per group). Scale bar = 25 μm; * *p* < 0.05, ** *p* < 0.01 vs. HFD-con by unpaired *t*-test.

The cross-sectional area (CSA) of the soleus muscle was only reduced by 12% in the HFD-con group, but this reduction was not observed in the melatonin and/or exercise training groups (Figure 2A,B). In contrast, the extramyocyte space of the soleus muscle in the HFD group was 20% larger than that in the P8ND group, and this increase was mitigated by the melatonin and/or exercise training interventions (Figure 2A,C). The number of muscle fibers in the soleus muscle was lower in the HFD-con group than that in the P8ND group, and this effect was mitigated in the melatonin and combined intervention groups but not in the exercise group (Figure 2A,D). Despite observing a trend towards deleterious effects of HFD on the morphology of the soleus muscle, the lack of statistically significant differences between groups could be attributed to high interindividual variation. The size and number of WG muscle fibers and the associated extramyocyte space were not significantly different between groups (Figure 2E–H).

### 2.3. Effects of Melatonin and Exercise Training on Molecular Mechanisms Associated with Sarcopenia

**Figure 3:**
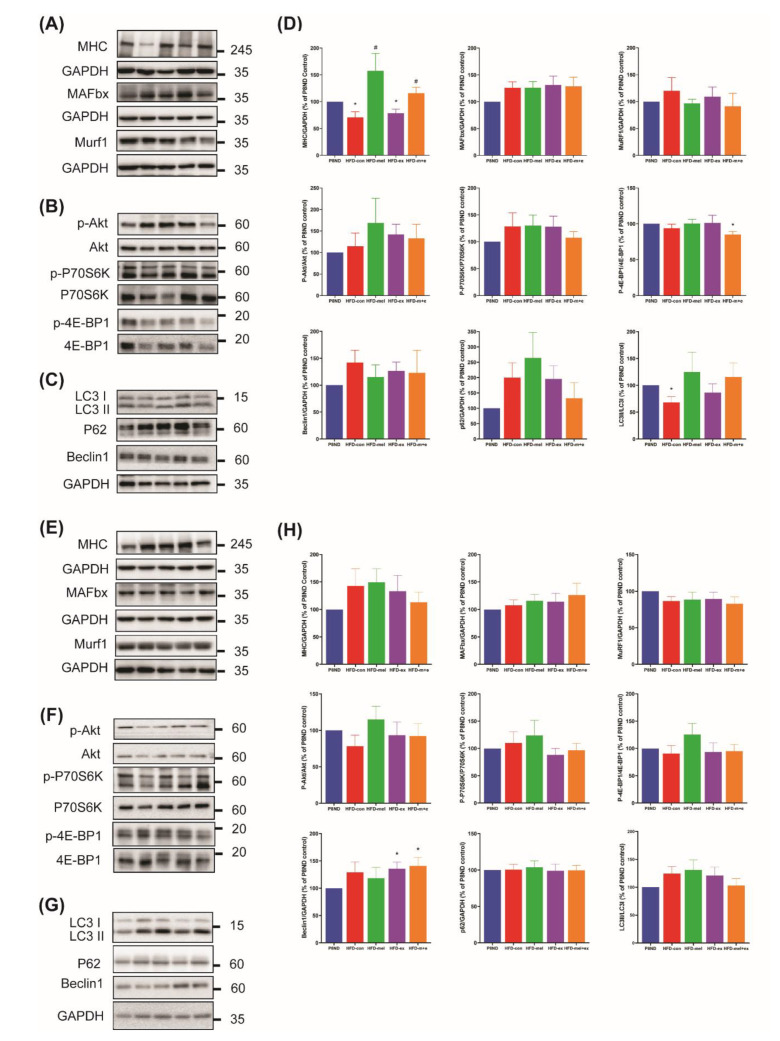
Effects of melatonin and exercise training on protein expression contributing to the molecular mechanisms associated with sarcopenia in the (**A**–**D**) soleus muscle and (**E**–**H**) white gastrocnemius muscle. Representative immunoblots of (**A**,**E**) MHC, MAFbx, and MuRF1, (**B**,**F**) the P-Akt/P-P70S6K/P-4EBP1 pathway, and (**C**,**G**) Beclin1/LC3I/II/P62 protein expression in the soleus muscle and white gastrocnemius muscle, respectively. (**D**,**H**) Graphs illustrate the densitometric analysis of protein levels, which were normalized to GAPDH or total form. Data are the mean ± SD (n = 6–10 mice per group); * *p* < 0.05 vs. P8ND by one-sample *t*-test, and ^#^ *p* < 0.05 vs. HFD-con by unpaired *t*-test.

Next, we examined the levels of key proteins involved in regulating skeletal muscle mass and function, including those involved in protein degradation, such as muscle ring finger 1 (MuRF1) and muscle atrophy F-box (MAFbx)/atrogin-1, the protein synthesis (Akt-P70-S6K/4EBP1) pathway, and autophagy. In the soleus muscle, we observed a significant reduction in myosin heavy chain (MHC) in the HFD-con group compared to the P8ND group, which was prevented by melatonin or the combined intervention, but not by exercise alone. However, the MHC levels in the WG were similar across all groups. In addition, the expression of MAFbx or MuRF1 was not significantly increased in either the soleus or WG muscles from the HFD-con group compared to the P8ND group (Figure 3A,D,E,H). We also examined the phosphorylation status of key proteins involved in protein synthesis and translation initiation signaling pathways, including Akt, P70S6K, and 4EBP1. There were no significant differences in the levels of phosphorylated forms of these proteins in either the soleus or WG muscles from the HFD-con and P8ND groups, although there was high interindividual variation in the levels of phosphorylation (Figure 3B,D,F,H).

To assess autophagy pathway components, we examined the levels of Beclin 1—a key regulator of autophagosome formation—and the ratio of LC3II/I and P62 in both types of muscle. The level of Beclin 1 was higher in the WG muscle from the exercise and combination groups than in the P8ND group, but it was not significantly different in the soleus muscle across the groups. Interestingly, the LC3II/I ratio in the soleus muscle of HFD-con was significantly lower than that of P8ND,with a trend for attenuation of this change in the HFD-mel and HFD-m+e groups. There were no significant differences in the LC3II/I ratio in WG muscle across the groups. The levels of P62 in both types of muscle were not significantly different across the groups (Figure 3C,D,G,H).

We also assessed mitochondrial function by measuring O_2_ respiration and the H_2_O_2_ emission rate, but we did not observe significant differences between the groups, although there was a trend towards positive effects of melatonin and exercise. However, in both types of muscle, the mitochondrial calcium retention capacity in the HFD-con group was significantly lower than that of the P8ND group, and this reduction was significantly attenuated by melatonin and/or exercise interventions (Figure S2).

### 2.4. Inhibition of SC Senescence by Melatonin and Exercise

**Figure 4:**
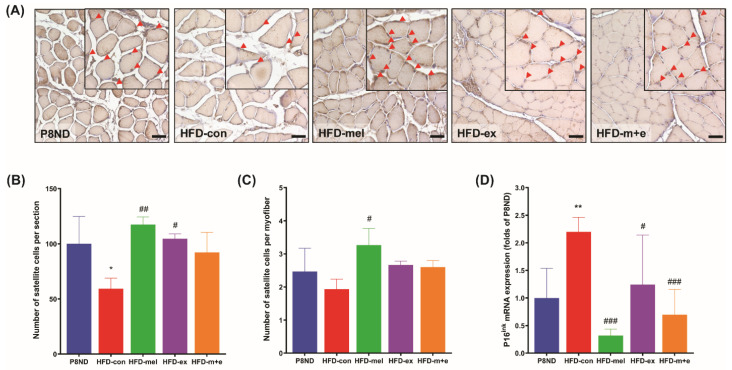
Melatonin and exercise training protected against obesity-induced deterioration of satellite cells’ population and senescence: (**A**) Representative Pax7-positive SC numbers (brown) in each group. Scale bar = 25 μm at 40× magnification; arrowheads indicate SCs. Bar graphs illustrating (**B**) the number of SCs per section (n = 3 mice per group), (**C**) the number of SCs per myofiber (n = 3 mice per group), and (**D**) the relative mRNA expression of p16^ink^ (n = 6 mice per group). Data are the mean ± SD; * *p* < 0.05 and ** *p* < 0.05 vs. P8ND; ^#^ *p* < 0.05, ^##^ *p* < 0.01, and ^###^ *p* < 0.001 vs. HFD-con using unpaired *t*-tests.

We observed structural abnormalities of the soleus muscle in the HFD-con group that were ameliorated by melatonin and/or exercise (Figure 2). Additionally, we investigated the impacts of melatonin and exercise training on the SC population in HFD-fed SAMP8 mice. Pax7+ was used as a marker to identify quiescent SCs in the soleus muscle [18]. The proportion of quiescent SCs per section was significantly lower in the HFD-con group (68.67% of P8ND), but this reduction was significantly attenuated by melatonin or exercise (Figure 4A,B). Although the number of SCs per myofiber was numerically lower in the HFD-con group than in the P8ND group, only the melatonin intervention group exhibited a significantly higher number of SCs per myotube than the HFD-con group (Figure 4C). Interestingly, the significantly higher levels of mRNA expression of the cellular senescence marker p16ink in the HFD-con group than in the P8ND group were significantly reduced by melatonin and/or exercise (Figure 4D).

**Figure 5:**
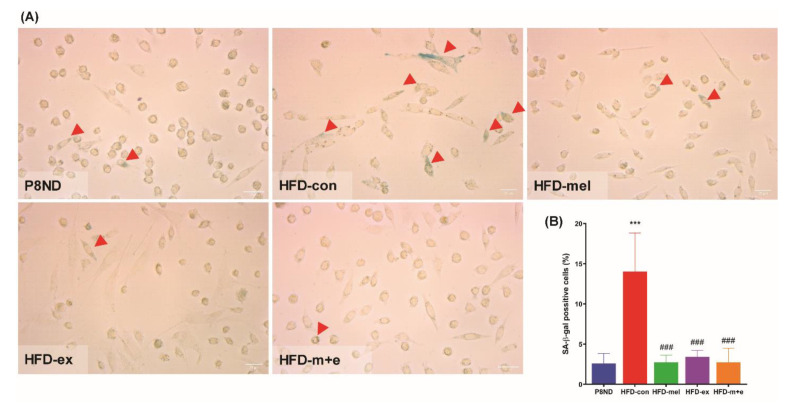
Senescence-associated β-galactosidase (SA-β-gal) in SC-derived primary myoblasts: (**A**) Representative SA-β-gal staining images illustrating SA-β-gal-positive cells (blue) in SC-derived primary myoblasts, and (**B**) the percentage of SA-β-gal-positive cells. Data are the mean ± SD (n = 6 mice per group). Scale bar = 25 μm; arrowheads indicate SA-β-gal-positive cells; *** *p* < 0.001 vs. P8ND and ^###^ *p* < 0.001 vs. HFD-con by unpaired *t*-test.

In primary myoblasts derived from SCs of each group, we consistently observed a higher percentage of SA-β-gal-positive cells in the HFD-con group than in the P8ND group (Figure 5A,B). At the same passage of culture, the percentage of SA-β-gal-positive cells was significantly lower in the HFD-mel, HFD-ex, and HFD-m+e groups than in the HFD-con group. Furthermore, immunofluorescence staining and immunoblot analysis revealed a higher expression of p16^ink^ in the HFD-con group than in the P8ND group, which was attenuated in the treatment groups (Figure S3).

### 2.5. Melatonin and Exercise Mitigate the HFD-Induced Deterioration of SC-Derived Myoblasts’ Proliferation and Differentiation Capacity

**Figure 6:**
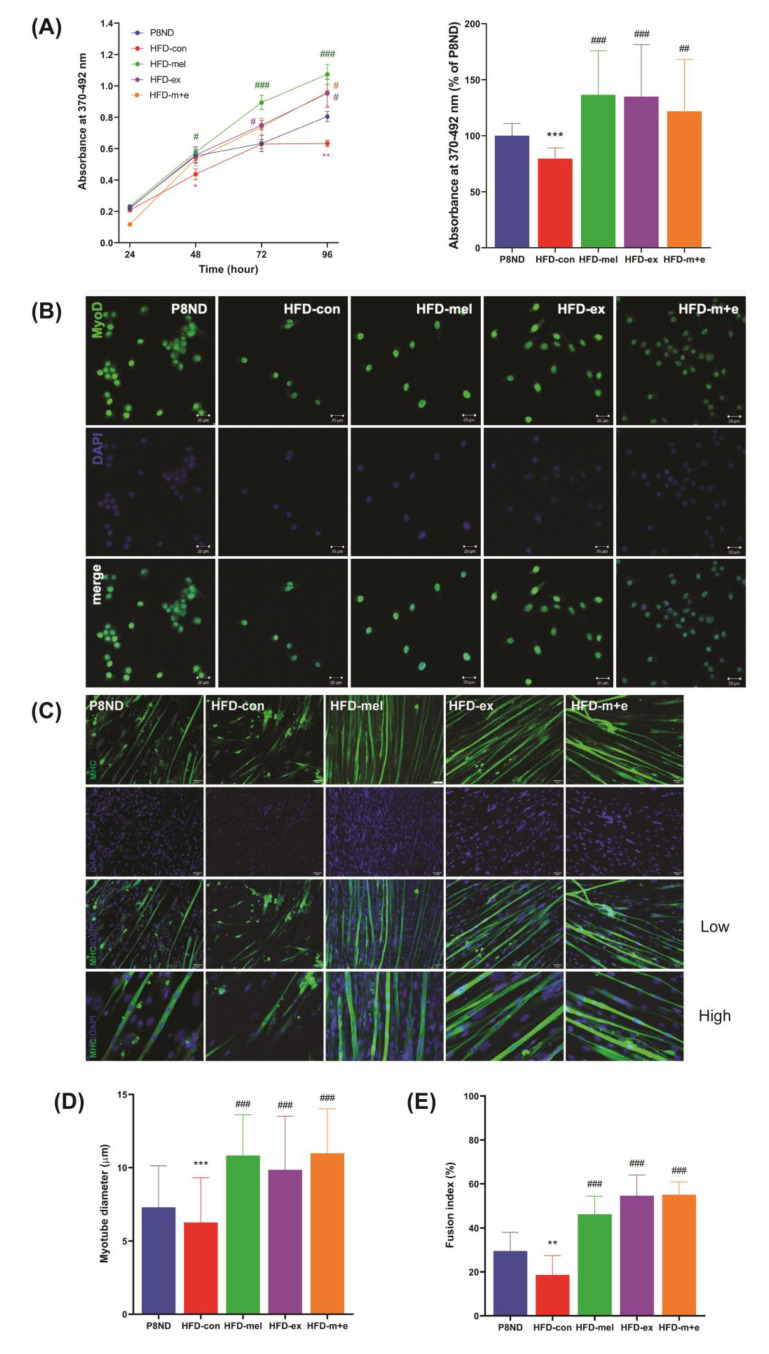
Melatonin and/or exercise training inhibited the HFD-induced deterioration of SC-derived myoblasts’ proliferation and differentiation capacity: (**A**) Cell proliferation capacity was measured by BrdU assay. (**Left**) BrdU labeling was measured during proliferation for 96 h. Data are the mean ± SD (n = 3 mice per group); * *p* < 0.05, ** *p* < 0.01 vs. P8ND; ^#^ *p* < 0.05, ^###^ *p* < 0.001 vs. HFD-con, using a two-way ANOVA and Dunnett’s multiple comparisons test. (**Right**) Bar graph illustrating the absorbance at 96 h after proliferation; *** *p* < 0.001 vs. P8ND; ^##^ *p* < 0.01 and ^###^ *p* < 0.001 vs. HFD-con, by unpaired *t*-test. (**B**) The number of proliferating cells after 72 h was determined by immunofluorescence. A representative immunofluorescent image of MyoD staining detected by a species-specific antibody conjugate with Alexa Fluor 488 (green) and DAPI (blue) nuclear staining is shown. Scale bar = 20 μm. (**C**) SC-derived primary myoblasts were induced to differentiate for 7 days and stained with an antibody for MHC, followed by a species-specific antibody conjugate with Alexa Fluor 488 (green) and DAPI for DNA (blue). Scale bar = 50 μm. Images with both low and high magnification were merged and displayed. (**D**) Myotube diameter and (**E**) fusion index were calculated. Data are the mean ± SD (n = 3 mice per group); ** *p* < 0.01 and *** *p* < 0.001 vs. P8ND; ^###^ *p* < 0.001 vs. HFD-con, by unpaired *t*-test.

A plausible explanation for the lower number of SCs in the HFD-con group than in the P8ND group is that obesity impairs SCs’ proliferation through cellular senescence. We observed a significantly lower cell proliferation capacity of primary myoblasts from the HFD-con group compared to the P8ND group (Figure 6), and this decreased proliferative activity was improved in the HFD-mel, HFD-ex, and HFD-m+e groups (Figure 6A). In addition, MyoD immunofluorescence after 72 h of myoblast proliferation indicated a lower number of cells in the HFD-con group than in the P8ND group, which was recovered by melatonin and/or exercise interventions (Figure 6B). Regarding differentiation capacity, we found that the diameter of differentiated myotubes and the fusion index were significantly lower in the HFD-con group than in the P8ND group, but they were normalized in the treated groups (Figure 6C–E). In addition, an in vitro study using H_2_O_2_-induced senescence of C2C12 cells consistently indicated that melatonin mitigates the H_2_O_2_-induced increase in SA-β-gal-stained myoblasts and the impairment of differentiation (Figure S4).

## 3. Discussion

Physically inactive elderly people suffer from accelerated sarcopenia and increased fat deposition, which may act additively to produce adverse outcomes [19]. Several studies suggest that melatonin and exercise training may play significant roles in preserving skeletal muscle mass and function, potentially preventing sarcopenia [13,20]. In this study, we observed differential effects of HFD and aging on the mass of different types of skeletal muscle. Typically, in aged skeletal muscle, atrophy occurs preferentially in type II fast-twitch muscle fibers compared with antigravity type I slow-twitch fibers [21], but in obesity the preference shifts from type II to type I muscle fibers [22]. One possible explanation for our findings is that aged and HFD-induced SO mice may compensate for changes in specific muscle fiber types by inducing chronic overload in antigravity muscles, regardless of age [23]. Nevertheless, muscle strength is considered to be a better indicator of sarcopenia than low muscle mass, as emphasized by the EWGSOP 2 [24]. In fact, as a model of SO, HFD-fed SAMP8 mice displayed a reduction in muscle strength, which was accompanied by microscopic structural changes, decreased MHC expression, decreased SC population, and senescence of SC-derived myoblasts, with lower proliferation and differentiation capacity in the soleus muscle. Although we did not observe a clear correlation between gross weight and microscopic findings, our results suggest that obesity in aged mice accelerates skeletal muscle dysfunction and microscopic deterioration of type I muscle, and that melatonin and exercise can attenuate the type I soleus muscle dysfunction. In accordance with our results, melatonin can preserve muscle fiber size in the soleus muscle in adiposity-increasing castrated rats [25]. In addition, mitochondrial dysfunction, which is a target for interventions against sarcopenia [26], was partially recovered by melatonin or exercise. Mitochondrial calcium retention capacity is not only associated with programmed cell death but is also correlated with skeletal muscle mass and function. Therefore, our results support the idea that melatonin and/or exercise can attenuate skeletal muscle dysfunction in HFD-fed aged SAMP8 mice—at least in part—by improving mitochondrial calcium retention capacity. The expression levels of MuRF1 and MAFbx in aged and young skeletal muscle are not consistent [27]. These observations, along with our results (Figure 3A,E), highlight how the loss of muscle mass during aging and obesity is mechanistically different in various models.

We rigorously evaluated the possible molecular mechanisms of sarcopenic phenotypes in our model [28], but we did not observe any obvious differences across the groups, which displayed high interindividual variability. SAMP8 is a phenotypically selected mouse strain, which may result in more variation in genetic background than in mice with a gain or loss of a specific gene. Therefore, our molecular analyses with high interindividual variability suggest that further studies are needed to clarify the molecular mechanisms of models of SO. Other possible causes of the high interindividual variability in our model include the duration of the diet and/or intervention or the age of the mice studied. We euthanatized the SAMP8 mice at 32 weeks of age, when sarcopenia may not be clearly established [29,30]. 

Our results regarding SCs in skeletal muscle are consistent with previous studies that reported that the lower number and function of SCs is an important mechanism of aging- [30,31] or obesity-induced [6] sarcopenia. Our study provided clear evidence that melatonin and exercise training mitigated the SO-induced decrease in SCs’ population and function. Even though evidence suggests that a lifelong reduction in the SC population might lead to age-related muscle fibrosis rather than accelerated sarcopenia [32], we cannot exclude the association between geroconversion of SC and sarcopenia [31]. The cellular senescence marker p16^ink^ is strongly upregulated during cellular senescence [31,33] and, consistent with our observations, p16^ink^ was de-repressed in aged SCs [31]. Therefore, the decrease in the SC population may result from irreversible quiescence to senescence, at least in part. Senescence of SC-derived primary myoblasts from HFD-con mice may be associated with a deficiency in the SC pool and their proliferation and differentiation capacity, which was mitigated by melatonin and/or exercise training intervention. Elimination of p16^ink^-positive senescent cells restored the SC quiescence for regeneration [31]. Therefore, our results suggest that melatonin and/or exercise intervention may restore the SC population by mitigating the upregulation of p16^ink^ in the HFD-con group. Furthermore, our in vitro results using a H_2_O_2_-induced senescence model support the effect of melatonin in vivo to restore SC function. These lines of evidence suggest that therapeutic interventions aimed at eliminating senescent SCs may help delay sarcopenia and provide a novel mechanism of action for melatonin and exercise intervention against sarcopenia. 

Our study has several limitations. First, our SO model of HFD-fed aged SAMP8 mice does not fully represent the phenotype of SO in humans. Therefore, other in vivo models of SO should be examined in future studies. Second, non-sarcopenic, young control or senescence-resistant strains (e.g., the SAMR1 strain) were not included in our study, limiting the interpretation of our results with respect to molecular mechanisms. Therefore, further studies using young control and older mice with more obvious characteristics of sarcopenia and SO are needed. In addition, although we observed that senescence may be a cause of sarcopenia using C2C12 cells, additional models to confirm our main findings will be necessary. Third, although we employed the dose of melatonin and exercise protocols used in previous studies, further studies will be needed to find the optimal duration of intervention, dose of melatonin, and/or intensity of exercise for elucidating molecular mechanisms and decreasing interindividual variability. Finally, we did not address possible gender differences in SO, which warrant further study. Despite these limitations, our study has several strengths. Firstly, we characterized an in vivo SO model using an SAMP8 strain with HFD, providing valuable insight into the pathophysiology of sarcopenia and SO. Furthermore, our study highlights the importance of developing appropriate experimental models for SO research. Secondly, we evaluated the effects of melatonin and/or exercise intervention on the phenotypes of the SO model, contributing to the development of therapeutic interventions for sarcopenia. Finally, we provided molecular evidence that melatonin and/or exercise-induced inhibition of SC senescence is a key mechanism underlying the benefits against SO.

## 4. Materials and Methods

### 4.1. Animal Care and Intervention

All animal experiments performed were approved by the Institutional Animal Care and Use Committee of Inha University (IACUC approval number, INHA-190523-649, Incheon, Republic of Korea). The median lifespan of SAMP8 mice is 9.7 months, the onset time of sarcopenia is around 32 weeks [29], and 24-week-old SAMP8 mice are at the adult stage [34]. Male SAMP8 mice (age 24 weeks) fed with ND or HFD for 8 weeks were allocated into 5 groups (10 per group): (1) P8ND; (2) HFD-con; (3) HFD-mel; (4) HFD-ex; and (5) HFD-m+e. Mice in the intervention groups received ~10 mg/kg/day of melatonin in their drinking water and/or exercise training on a treadmill (Dual Treadmill, DJ-344, Daejong, Seoul, Republic of Korea) for an additional 8 weeks. Mice in the P8ND and HFD-con groups were considered sedentary. At 32 weeks, mice were euthanatized under anesthesia (see Supplementary Methods for details).

### 4.2. Measurement of Grip Strength and Mitochondrial Function

During the experiment, the mice were weighed every 2 weeks. Skeletal muscle strength was assessed by grip strength using an animal strength meter (Bioseb, Pinellas Park, FL, USA) at age 24 and 32 weeks. Mitochondrial function is critical to energy metabolism and cellular function. Therefore, we measured the oxygen respiration, H_2_O_2_ emission, and calcium retention capacity of type I (soleus) and II (white gastrocnemius) muscles at the end of the experiment, using a polarographic high-resolution respirometer (Oxygraph-2k: O2k, Oroboros, Innsbruck, Austria) (see Supplementary Methods for details). 

### 4.3. Histology and Immunohistochemistry

Muscle tissue (n = 3–5 per group) was fixed in 4% paraformaldehyde, embedded in paraffin, and processed for hematoxylin and eosin staining for assessment of the cross-sectional area (CSA), extramyocyte space, and number of muscle fibers. Histological data were assessed using ImageJ software (NIH, Bethesda, MD, USA). For the immunohistochemical detection of SCs, paired box protein 7 (Pax7) expression in the muscle was identified using anti-Pax7 antibody (dilution 1 μg/mL, Novusbio, NBP2-44588), according to the manufacturer’s instructions. An image of each specimen was recorded under a bright-field microscope (Carl Zeiss, Jena, Germany).

### 4.4. Western Blot Analysis

Protein was extracted in radioimmunoprecipitation assay buffer, containing a protease inhibitor and phosphatase inhibitor cocktail (Merck, Darmstadt, Germany). Total protein was subjected to Western blotting using appropriate antibodies (Supplementary Table S1), as described in [35]. Briefly, the isolated protein was separated by sodium dodecyl sulfate–polyacrylamide gel electrophoresis and transferred to a PVDF membrane. After incubation in 5% skimmed milk for 1 h, the membrane was incubated with primary antibodies, followed by an HRP-conjugated goat anti-rabbit IgG or goat anti-mouse IgG antibody. Immunoreactivity was detected with an enhanced chemiluminescence detection kit (Merck Millipore, Temecula, CA, USA) and visualized using the Chemi-Doc System (Bio-Rad Laboratories, Hercules, CA, USA). The signal intensity of each protein band was determined by densitometry, using ImageLab software (Bio-Rad Laboratories).

### 4.5. Culture of SC-Derived Primary Myoblasts and C2C12

SC-derived primary myoblasts were isolated according to a previously described method [36], with slight modifications. Tibialis anterior (TA) muscles were digested, and a filtered cell mixture was collected. Cells were incubated in a non-coated dish for 1 h, followed by pre-plating 3 times on a gelatin-coated dish. Purified myoblasts were cultured in Ham’s F10 medium supplemented with 20% fetal bovine serum (FBS). Detailed procedures for primary myoblast isolation and culture are described in the Supplementary Methods.

C2C12 murine myoblasts (American Type Culture Collection; Manassas, VA, USA) were cultured in Dulbecco’s Modified Eagle’s Medium (DMEM; Gibco, Grand Island, NY, USA) supplemented with 100 U/mL of penicillin, 100 μg/mL of streptomycin, and 10% heat-inactivated FBS in a humidified atmosphere of 5% CO_2_ at 37 °C. For differentiation of C2C12, the differentiation medium (DMEM with 2% horse serum) was renewed every 24 h for 4 days. 

### 4.6. BrdU Cell Proliferation Assay

A bromodeoxyuridine (BrdU) assay to evaluate cell proliferation was conducted according to the manufacturer’s instructions (Merck). Briefly, cells (1 × 10^4^ cells/well) were seeded into 96-well plates, followed by culture for the indicated times. BrdU (10 μM) was added to the cells and incubated for 24 h. The labeling medium was discarded, and FixDenat solution (200 μL/well) was added and incubated for 30 min to allow DNA degeneration. Subsequently, the cells were incubated with anti-BrdU-POD antibody (100 μL/well) for 1.5 h, and then rinsed 3 times with phosphate-buffered saline (PBS). After washing, a chromogenic tetramethylbenzidine solution was added, and the cells were incubated for 5 min at room temperature. The absorbance at 370 nm (reference at 492 nm)—directly related to the proliferation rate of the cells—was measured. In addition, we evaluated the number of cells proliferating after 72 h by measuring the immunofluorescence of an anti-MyoD antibody detected by a species-specific antibody conjugate with Alexa Fluor 488 (green) and DAPI (blue) nuclear staining. 

### 4.7. Evaluation of Differentiation Capacity of Primary Myoblast

SC-derived primary myoblasts were cultured on glass coverslips in 12-well plates. The SC-derived primary myoblasts were maintained in the proliferation medium described above until their level of confluence reached 90% (day 0). The growth medium was then replaced with DMEM containing 5% horse serum and renewed daily for 7 days. Thereafter, the differentiated myotubes were analyzed by immunofluorescence staining using an anti-myosin heavy chain antibody (anti-MHC) conjugated with Alexa Fluor 488 and DAPI to measure the myotube diameter and fusion index (Supplementary Methods), and then visualized at 20× magnification using a fluorescence microscope (DP80; Olympus, Tokyo, Japan). 

### 4.8. Senescence-Associated β-Galactosidase (SA-β-Gal) Staining and mRNA Expression of the Cdkn2a (P16^ink^) Gene

SA-β-gal staining was performed to determine the senescence phenotype of SC-derived primary myoblasts. Cells (1 × 10^5^ cells/well) from each group were grown in a 12-well plate overnight. The cells were then washed with PBS twice, fixed with 3.7% formaldehyde in PBS for 5 min at RT, and washed with PBS twice. Subsequently, the cells were incubated with an SA-β-gal staining solution (0.5 mL/well) for 24 h. The reaction was stopped by replacing the staining solution with PBS. Stained cells were examined under a light microscope (Leica Microsystems, Wetzlar, Germany), and the percentage of SA-β-gal-positive cells was calculated. The experiments were performed with at least three cell donors in duplicate. The total RNA (2 μg) extracted using TRIzol reagent (Ambion, Life Technologies, Carlsbad, CA) and chloroform was converted to cDNA using a cDNA synthesis kit (Takara Bio, Shiga, Japan). Quantitative real-time PCR (qRT-PCR) was conducted using a CFX96 Touch Real-Time PCR machine (Bio-Rad Laboratories) following amplification reactions containing 10 μL of 2x iTaq Universal SYBR Green Supermix (Thermo Fisher Scientific, Waltham, MA; antibody-mediated hot-start iTaq DNA polymerase, dNTPs, MgCl_2_, SYBR Green I dye, enhancers, stabilizers, and blend of passive reference dyes), 0.5 μL of each forward (GCC CAA CGC CCC GAA CTC TTT C) and reverse (GCG ACG TTC CCA GCG GTA CAC A) primer, 7 μL of DNase-free water, and 2 μL of cDNA. The relative gene expression was calculated by the value of comparative cycle threshold (Ct) and normalized with GAPDH as a housekeeping gene. Gene expression was analyzed using CFX manager software. The fold of relative gene expression was determined for the control group after normalization using the 2^−ΔΔCt^ method.

### 4.9. Statistical Analysis

Data are presented as the mean ± standard deviation (SD), and they were analyzed for statistical significance using Prism (v.9; GraphPad Software, San Diego, CA, USA). Groups were compared using the paired *t*-test, unpaired *t*-test, or two-way analysis of variance (ANOVA) followed by Dunnett’s post hoc test, as appropriate. Outlier data were identified by the Grubbs method and removed from the analysis. When comparing the band intensities of immunoblots from each group with those from the P8ND control group, a one-sample *t*-test was used. Statistical difference was accepted as significant at *p* < 0.05.

## 5. Conclusions

Our study revealed that melatonin and exercise training were effective in mitigating sarcopenia in an SO animal model of HFD-fed, aged SAMP8 mice, as evidenced by the improvements in muscle mass and strength. Mechanistically, the anti-senescence effects of melatonin and exercise training were found to preserve the SC pool by enhancing the proliferation and differentiation capacities of SCs in skeletal muscle. Furthermore, these interventions were also shown to improve mitochondrial function. Taken together, our findings suggest that targeting the anti-senescence effects of melatonin and exercise on SCs in skeletal muscle may be a promising therapeutic approach for mitigating SO. Further validation studies in other models and humans will be necessary to confirm our results. 

## Data Availability

Not applicable.

## Supplementary Materials

The following supporting information can be downloaded at: https://www.mdpi.com/article/10.3390/ijms24076097/s1.
