# Supplementary material for: Melatonin and Exercise Counteract Sarcopenic Obesity through Preservation of Satellite Cell Function
Source: Int J Mol Sci. 2023 Mar 23;24(7):6097. doi: 10.3390/ijms24076097 (PMC10094434; doi:10.3390/ijms24076097)
Supplement: Supplementary file 1 [file ijms-24-06097-s001.zip › Supplementary Figures.pdf]

Supplementary Figures

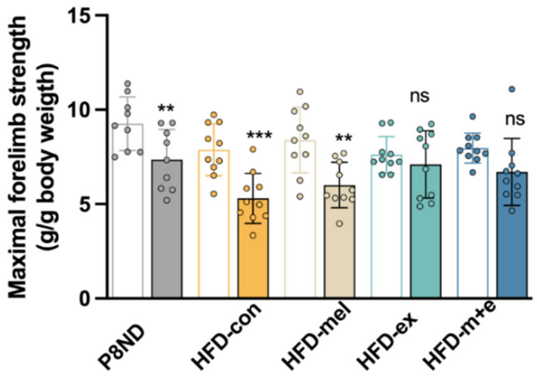

Figure S1. Comparison of maximal forelimb strength between before (open bars; 6 months) and after (filled bars; 8 months) intervention. Data are mean ± SD, n = 9–10; ** $p < 0.01$, *** $p < 0.001$ vs. before intervention by paired *t*-test. ns, not significant.

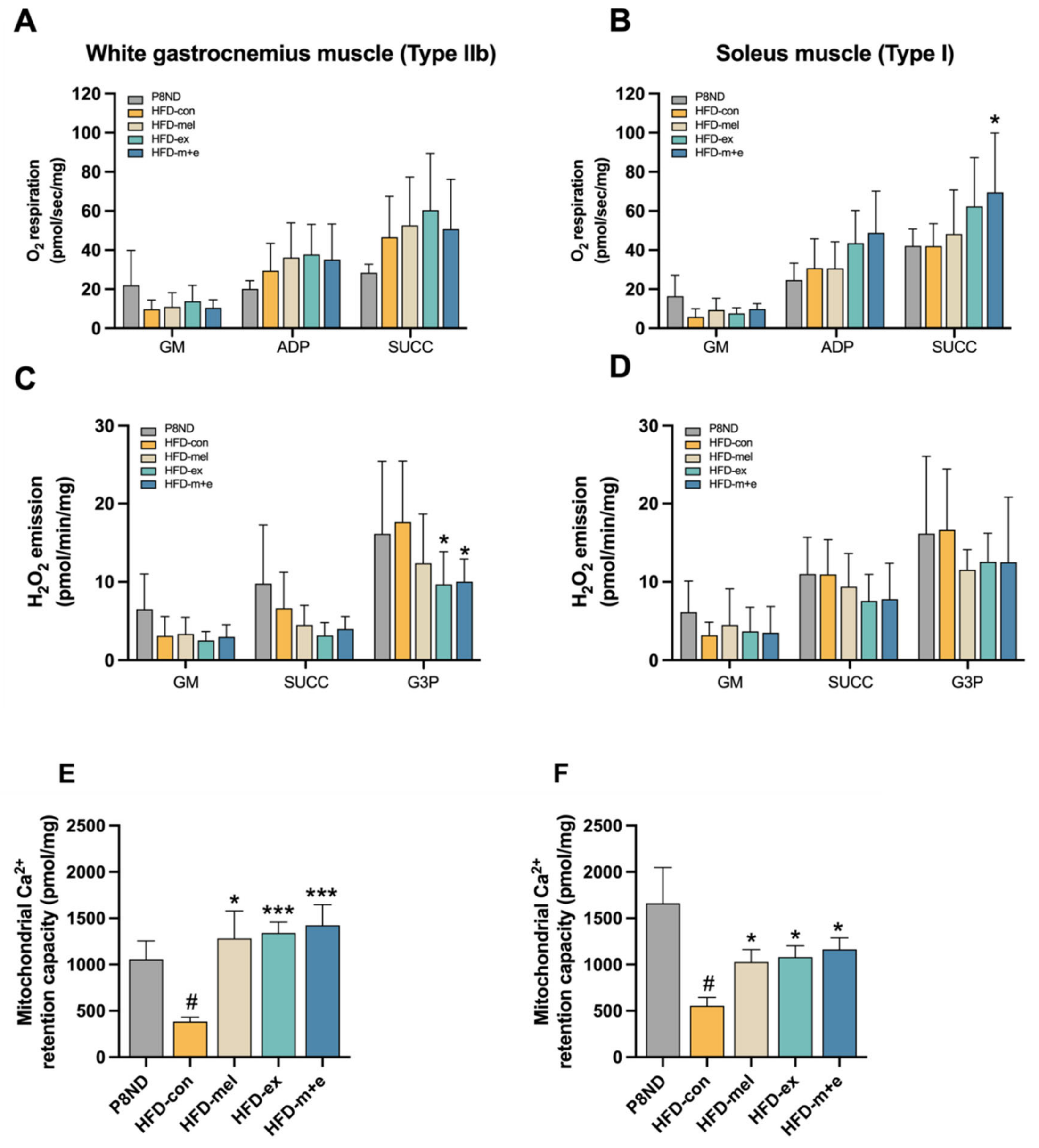

Figure S2. Effects of melatonin and/or exercise training on mitochondrial function. Mitochondrial function for (**A**,**B**) O_2 respiration, (**C**,**D**) H_2O_2 emission, and (**E**,**F**) calcium retention capacity were measured in white gastrocnemius (**A**,**C**,**E**) and soleus muscle (**B**,**D**,**F**), respectively (n = 9–10). Data presents mean ± SD. * $p < 0.05$, ** $p < 0.01$, *** $p < 0.001$ compared with HFD-con and $^{\#}$ $p < 0.05$ versus P8ND group by unpaired t-test. GM, glutamate/malate; ADP, adenosine diphosphate; SUCC, succinate; G3P, glycerol-3-phosphate.

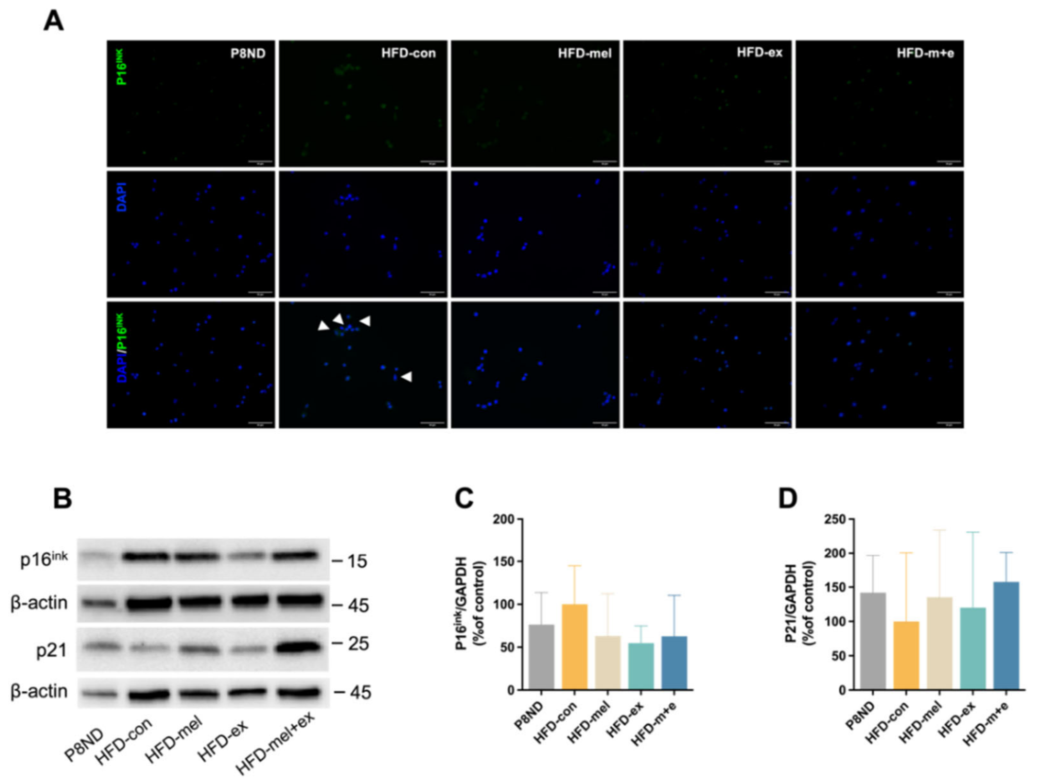

Figure S3. Expression of cellular senescence markers, $p16^{ink}$ in SC-derived primary myoblasts. (**A**) Representative immunofluorescence shows $p16^{ink}$ (green) and nucleus (DAPI; blue), and (**B**) immunoblots for $p16^{ink}$ and p21 protein expression. (**C**,**D**) Density analyses of (**C**) $p16^{ink}$ and (**D**) p21 protein expression are shown. Data are mean ± SD, n = 3–5. Scale bar represents 50 μm. White arrowheads indicate $p16^{ink}$ positive cells.

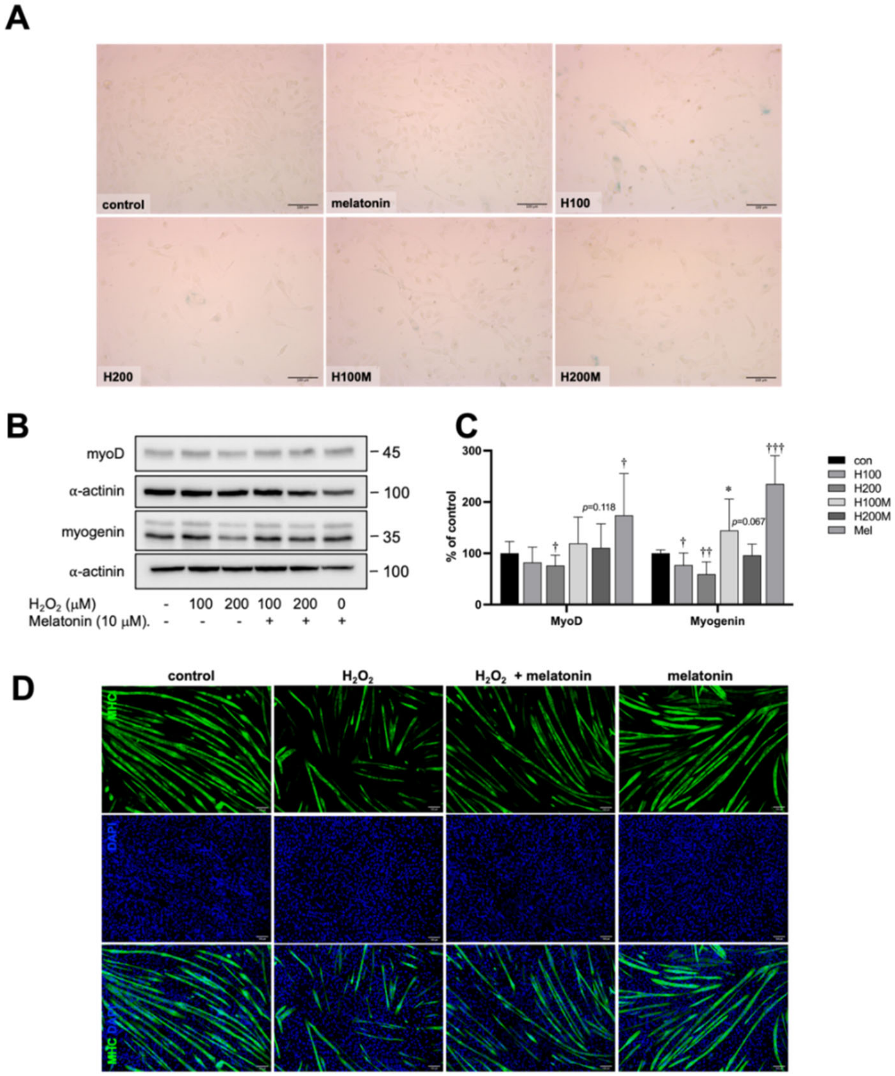

Figure S4. Effect of melatonin on myoblast senescence and deterioration of myotube differentiation by H_2O_2. (**A**) Representative image of SA-β-gal staining of myoblast and (**B**) immunoblots for myoD and myogenin in C2C12 myoblasts with or without treatment of H_2O_2 and/or melatonin are shown. (**C**) Bar graph indicates the density analysis of band intensities from immunoblots. The data are expressed as mean ± SD of 3 independent experiments. $^{\dagger}p < 0.05$, $^{\dagger\dagger}p < 0.01$, and $^{\dagger\dagger\dagger}p < 0.001$ vs. untreated control, and $^{*}p < 0.05$ or marginal significant p-values for H_2O_2+melatonin treatment vs. H100 by unpaired t test. Con, vehicle-treated control; H100, cells treated with H_2O_2 at 100 μM; H200, cells treated with H_2O_2 at 200 μM; H100M, cells treated with H_2O_2 at 100 μM and melatonin (10 μM); H200M, cells treated with H_2O_2 at 200 μM and melatonin; Mel, cells treated with melatonin

only. (**D**) Representative immunofluorescent image using anti-MHC antibody followed by fluorescent staining with species-specific Alexa Fluor 488 conjugated antibody (green) and DAPI (blue) nucleus staining is shown. Scale bars represent 100 μm.
