# Supplementary material for: Melatonin and Exercise Counteract Sarcopenic Obesity through Preservation of Satellite Cell Function
Source: Int J Mol Sci. 2023 Mar 23;24(7):6097. doi: 10.3390/ijms24076097 (PMC10094434; doi:10.3390/ijms24076097)
Supplement: Supplementary file 1 [file ijms-24-06097-s001.zip › Supplementary Methods_revision.pdf]

Supplementary Methods

1. *Animal care*

We used six-weeks-old male senescence-accelerated mice (SAMP8) obtained from Central Lab Animal (Seoul, Republic of Korea). They were housed individually and maintained under controlled light conditions (12:12 h, light/dark cycle) at an ambient temperature of 22 ± 2 °C. Laboratory diet and tap water were provided ad libitum throughout the study. Mice at 16 weeks of age were allocated to two groups, including 10 mice for a normal diet (ND) and 40 mice on the 60% high fat diet (HFD; Research Diets Inc., New Brunswick, NJ, USA). At 24 weeks of age, they were divided into 5 groups (n=10 animals/group) as follows: (1) P8ND, SAMP8 ND control group; (2) HFD-con, SAMP8 HFD control group; (3) HFD-mel, SAMP8 HFD + melatonin group; (4) HFD-ex, SAMP8 HFD + exercise group; (5) HFD-m+e, SAMP8 HFD + melatonin + exercise group. During the experiment, body weight (BW) and physical performance were measured. At 32 weeks of age, mice were sacrificed under anesthesia using tiletamine hydrochloride and zolazepam hydrochloride (Zoletil), 30 mg/kg BW, and cardiac blood and tissues were collected.

2. *Melatonin administration*

For melatonin treatment, 24-weeks-old mice of HFD-mel and HFD-m+e groups received approximately 10 mg/kg/day of melatonin in drinking water for 8 weeks. Melatonin was dissolved in ethyl alcohol to increase the solubility and diluted in drinking water at a final concentration of 0.066%. Mice in the P8ND, HFD-con and HFD-ex groups received the same amount of ethyl alcohol in drinking water (0.066%). Drinking water with and without

melatonin was covered with aluminum foil for light protection and changed twice a week. The stability of melatonin in drinking water measured by UV-visible spectrophotometer at 3 and 7 days after dissolution was 90.7% and 90.3% (data not shown).

3. *Exercise regimen*

Six-months-old mice in the exercise group with and without melatonin supplement were familiarized to exercise on a treadmill running machine (Dual treadmill, DJ-344; Daejong, Seoul, South Republic of Korea) for 5 consecutive days before the main exercise training. After familiarization, mice ran on the treadmill running for 5 days per week for 8 weeks. The exercise training protocol started at an initial speed of 8 m/min for 5 min, and then mice ran with speed of 12 m/min for 35 min.

4. *Fusion index and myotube diameter assessment*

The immunofluorescence image with MHC and DAPI staining was used to examine the myotube diameter and fusion index. The diameter of the myotube was calculated as the average of 10 measurements along the myotube length for representative measure using the Image J program. The fusion index was calculated as the number of nuclei incorporated into myotubes expressed as a percentage of the total number of nuclei in the image following the formula below.

$$\text{\% Of fusion index} = \frac{number\ of\ nuclei\ in\ myotube}{number\ of\ nuclei\ present\ in\ a\ given\ field} \times 100$$

5. *Measurement of mitochondrial function*

Type I (Soleus) and Type II (White gastrocnemius) muscle was rapidly collected, weighed and placed in ice-cold PBS. Following the preparation of permeabilized myofibers using saponin (50 μg/mL), mitochondrial O_2 respiration was measured using polarographic high-resolution respirometry (Oxygraph-2k, Oroboros, Innsbruck, Austria) at 30°C in assay buffer (buffer Z with 50 μM EGTA and 20 mM creatine) under the following addition: (1) 5 mM glutamate (complex I substrate) and 2 mM malate (complex I substrate), (2) 4mM ADP (state 3 condition), (3) 10 mM succinate (complex II substrate), and (4) 10 μg/mL oligomycin (inhibitor of mitochondrial ATP synthase). The mitochondrial O_2 respiration is expressed as pmol/sec per mg wet tissue weight. Mitochondrial H_2O_2 emission was analyzed in buffer Z [in the presence of Amplex Red at 37°C (ΔF/ min) with magnetic stirrer] during state 4 respiration (10 μg/mL oligomycin) by monitoring oxidation of Amplex Red (excitation/emission wavelengths λ = 568/581 nm) using a SPEX FluoroMax 4 spectrofluorometer (HORIBA Jobin Yvon, Edison, NJ, USA) under the following protocol: (1) 5 mM glutamate + 2 mM malate, (2) 10 mM succinate, and (3) 10 mM glycerol-3 phosphate (lipid substrate). The mitochondrial H_2O_2 emission rate following the deletion of value from each of the standard values (standard curve) was measured from the slope of Δ*F*/min gradient values and expressed as pmol/min per mg wet tissue weight. We measured calcium retention capacity by measuring overlaid traces of changes in Ca^{2+}-stimulated fluorescence (ΔF/ min) with Calcium Green-5N at 37°C during state 4 condition using a SPEX FluoroMax 4 spectrofluorometer. After establishing background ΔF in buffer Z containing (1 μM Calcium Green-5 N, 5 mM glutamate, 2 mM

malate, and 80 μM EGTA), the reaction was started by adding 30 μM Ca^{2+} pulses (excitation/emission wavelenghs = 506/532 nm). Calcium retention capacity before permeability transition pore opening is expressed as pmol/mg wet tissue weight.

6. *Primary SC-derive myoblast isolation and cell culture*

Minced tibialis anterior (TA) muscles were digested in solution consisting of 2.4 U/mL of dispase II (Roche Diagnostics, Mannheim, Germany), 1.5 U/mL of collagenase D (Roche Diagnostics), and 2.5 μM $CaCl_2$. After 1-hr incubation of digested mixture in CO2 incubator at 37°C, filtered cell mixture was centrifuged at 196 x *g* for 3 min at room temperature (RT). The supernatant was discarded, and the cell pellet was resuspended in 10 mL of Ham's F10 medium (Lonza Bioscience, Walkersville, MD, USA) supplemented with 20% newborn calf serum (NBCS, Gibco), 100 U/mL of penicillin, 100 μg/mL of streptomycin, and 5 ng/mL recombinant human fibroblast growth factor-basic (EMD Millipore, Darmstadt, Germany) in a non-coated dish for 1 hr, then pre-plated 3 times in a 0.1% gelatin-coated dish (Sigma Aldrich, St. Louis, MO, USA). Purified myoblasts were cultured in Ham's F10 supplemented with 20% fetal bovine serum (FBS) instead of NBCS to expand the number of purified myoblasts. We used the same passage number of different group donors during all experiments (cell passage number < 10). For the differentiation of primary myoblasts, when the myoblast reached 90% confluency, the growth medium was replaced with DMEM containing 5% horse serum and renewed daily for 7 days. Murine C2C12 myoblasts obtained from the American Type Culture Collection (Manassas, VA, USA) were cultured in Dulbecco's Modified Eagle's Medium (DMEM; Gibco, Grand Island, NY, USA) supplemented with 100 U/mL of penicillin, 100 μg/mL of

streptomycin and 10% heat-inactivated FBS in a humidified atmosphere of 5% CO_2 at 37°C. For the differentiation of C2C12 myoblasts, proliferating media was changed to differentiation media (DMEM with 2% horse serum) when the myoblasts reached ~100% confluency, and was renewed every 24 hrs for 4 days.
