# Supplementary material for: Melatonin and Exercise Counteract Sarcopenic Obesity through Preservation of Satellite Cell Function
Source: Int J Mol Sci. 2023 Mar 23;24(7):6097. doi: 10.3390/ijms24076097 (PMC10094434; doi:10.3390/ijms24076097)
Supplement: Supplementary file 1 [file ijms-24-06097-s001.zip › Supplementary Table_revision.pdf]

Supplementary Tables

Table S1. List of antibodies used in the western blotting analysis.

Antibodies	Source	Dilution	Cat. No. of manufacturer	MW (kDa)
MHC	Rabbit	1:1000	Ab91506	245
MAFbx	Mouse	1:1000	SC-166806	42
MuRF1	Mouse	1:1000	SC-398608	40
p-4EBP1	Rabbit	1:1000	CS#9459	15-20
4EBP1	Rabbit	1:1000	CS#9452	15-20
p-P70S6K	Mouse	1:1000	CS#9206	70/85
P70S6K	Rabbit	1:1000	CS#9202	70/85
p-Akt(Ser^{473})	Rabbit	1:1000	CS#9271	60
Akt	Rabbit	1:1000	CS#9272	60
LC3 I/II	Rabbit	1:1000	CS#12741	14/16
Beclin1	Rabbit	1:1000	CS#3738	60
p21	Rabbit	1:1000	CS#64016S	21
p16ink	Rabbit	1:1000	Ab211542	16
GAPDH	Rabbit	1:2000	Pro-Science 3781	37

Table S2. Effects of melatonin and/or exercise training on levels of fasting blood glucose and adipomyokines.

Parameter/Group	P8ND	HFD-con	HFD-mel	HFD-ex	HFD-m+e
**FBG (mg/dL)**	95.9 ± 21.3	244.8 ± 47.4***	173.1 ± 75.8$^{\#}$	179.0 ± 56.9$^{\#\#}$	136.3 ± 36.0$^{\#\#\#}$
**Adiponectin (ng/mL)**	8185 ± 981	6328 ± 1286**	6152 ± 2217	6823 ± 2075	6925 ± 1974
**Resistin (ng/mL)**	5.32 ± 1.20	10.0 ± 3.09***	8.94 ± 3.36	10.5 ± 2.90	10.4 ± 5.81
**Leptin (ng/mL)**	4.45 ± 2.95	19.5 ± 15.9*	10.0 ± 5.84	8.08 ± 5.84	8.54 ± 3.84
**TNF-α (pg/mL)**	7.45 ± 2.29	9.88 ± 3.14	8.22 ± 2.62	8.89 ± 3.26	9.60 ± 3.31
**BDNF (pg/mL)**	1.42 ± 1.26	0.54 ± 0.17	1.38 ± 1.20	0.95 ± 0.72	0.90 ± 0.72

Data are mean ± SD, n = 9–10, $^{*}p < 0.05$, $^{**}p < 0.01$, $^{***}p < 0.001$ vs. P8ND, $^{\#}p < 0.05$, $^{\#\#}p < 0.01$, $^{\#\#\#}p < 0.001$ vs. HFD-con by one-way ANOVA with Dunnett's post hoc test. FBG, Fasting Blood Glucose; TNF-α, Tumor Necrosis Factor-α; BDNF, Brain-Derived Neurotrophic Factor.
